# Antigen targeting and anti-tumor activity of a novel anti-CD146 ^212^Pb internalizing alpha-radioimmunoconjugate against malignant peritoneal mesothelioma

**DOI:** 10.1038/s41598-024-76778-z

**Published:** 2024-10-29

**Authors:** Kim Lindland, Marion Masitsa Malenge, Ruth Gong Li, Roxanne Wouters, Tina Bjørnlund Bønsdorff, Asta Juzeniene, Srdan M. Dragovic

**Affiliations:** 1 Department of Molecular Medicine, Institute of Basic Medical Sciences, University of Oslo, 0316 Oslo, Norway; 2 Department of Radiation Biology, Institute of Cancer Research, The Norwegian Radium Hospital, Oslo University Hospital, 0379 Oslo, Norway; 3 Oncoinvent ASA, 0484 Oslo, Norway; 4 Laboratory of Tumour Immunology and Immunotherapy, Department of Oncology, Leuven Cancer Institute, KU Leuven, 3000 Leuven, Belgium; 5 Department of Physics, University of Oslo, Oslo, Norway

**Keywords:** CD146 antigen, Targeted alpha therapy, Radioimmunoconjugate, ^212^Pb, Internalization, Malignant peritoneal mesothelioma xenograft model, Biophysics, Cancer, Drug discovery, Medical research, Oncology

## Abstract

Malignant mesothelioma, a highly aggressive cancer that primarily affects the serosal membranes, has limited therapeutic options, particularly for cavitary tumors, such as peritoneal and pleural malignant mesothelioma. Intracavitary administration of a radioimmunoconjugate to locally target mesothelioma cancer cells has been proposed as a treatment. CD146, upregulated in mesothelioma but not in healthy tissues, is a promising therapeutic target. This study characterized CD146 expression and binding/internalization kinetics of the CD146-targeting antibody OI-3 coupled with ^212^Pb (^212^Pb-TCMC-OI-3) in human mesothelioma cells. Flow cytometry showed that both chimeric (chOI-3) and murine (mOI-3) antibodies rapidly bound and internalized within 1–6 h in MSTO-211H cells. ^212^Pb-TCMC-chOI-3 exhibited 3.1- to 13.7-fold and 3.1- to 8.5-fold increased internalized ^212^Pb and ^212^Bi atoms per cell at 2 and 24 h, respectively, compared to isotype control, underscoring enhanced internalization efficiency. Intraperitoneal administration of ^212^Pb-TCMC-mOI-3 to mice with intraperitoneal MSTO-211H xenografts improved median survival by a ratio of 1.3 compared to non-binding ^212^Pb-TCMC-mIgG1. The ability of ^212^Pb-TCMC-mOI-3 to target and inhibit the growth of intraperitoneal mesothelioma xenografts supports targeted radionuclide therapy’s efficacy for metastatic peritoneal mesothelioma. This study highlights the potential of localized CD146-targeted radioimmunotherapy for malignant mesothelioma, offering a new avenue for improving patient outcomes.

## Introduction

Malignant mesothelioma (MM) primarily affects the serosal membranes, most commonly in the pleural and peritoneal cavities^1^. Current therapeutic modalities include surgery, radiation, and chemotherapy with pemetrexed and cisplatin, and immunotherapy with nivolumab and ipilimumab^1–4^. MM is difficult to treat and has a poor prognosis, with current therapies like chemotherapy and immune checkpoint inhibitors offering limited survival benefits with a median survival time of 9–17 months after diagnosis^2,5^. One encouraging therapeutic strategy involves the use of full-length antibodies labeled with short-lived radioactive isotopes that can specifically target CD146-expressing malignant mesothelioma cells. In this way, killing of cancer cells is accomplished while minimizing the damage to healthy neighboring tissues when radioimmunoconjugates (RIC) containing alpha emitters are delivered intracavitary, where MM develops^6,7^. A phase 1 study using intravenous administration of a monoclonal antibody (mAb) against another MM target, mesothelin, coupled with ^227^Th, was recently completed (NCT03507452). This study aimed to confirm the potential of this approach and evaluate the safety, tolerability, and preliminary efficacy of radioimmunotherapy (RIT) in MM patients.

Cluster of differentiation 146 (CD146), also known as MCAM, is a transmembrane glycoprotein^8^. This antigen plays a crucial role in various physiological processes including cell development, signal transduction, cell migration, mesenchymal stem cell differentiation, endothelial signaling, angiogenesis, and immune responses^8,9^. CD146 is upregulated in several cancer types, including melanoma, breast, prostate, ovarian, liver, lung, pancreatic, kidney, and mesothelioma^8,10,11^. Elevated CD146 expression has been correlated with increased metastatic capacity in several cancers, where high CD146 expression promotes cancer cell detachment and the development of distal metastases^9,10,12^. Therapeutic and diagnostic antibodies targeting CD146 (ABX-MA1, AA98, TsCD146, YY146, ME-9F1, etc.) have been previously reported^9,13–19^. A fully human ABX-MA1 antibody was evaluated for metastatic melanoma and osteosarcoma in preclinical studies^15,20^. A murine mAb has demonstrated effectiveness in inhibiting cancer progression in several xenograft tumors such as hepatocellular carcinoma, pancreatic cancer, and other cancers^21^. The rat TsCD146 antibody demonstrates specificity for cancer-associated CD146, detects cancer microparticles in patient plasma, and shows reactivity in both cancer patient biopsies and animal models; however, it does not bind to CD146 expressed in healthy tissues, including endothelial cells. Additionally, it has shown therapeutic effects by reducing the growth of CD146-positive cancer cells in animal models^22^. The YY146 antibody used in immunoPET imaging has high specificity and binding affinity for CD146-positive cancer cells, making it a valuable tool for CD146-targeted cancer detection and therapy monitoring^16,17,23,24^. ME-9F1 has been demonstrated to be a promising tool for improved tumor-specific drug delivery and has potential therapeutic applications in hepatocellular carcinoma and other liver tumors^25^. Therefore, CD146 may be used in targeted therapies for several types of cancers, including specific, targeted, and intracavitary radionuclide therapies^13,21,22,24,26–28^.

Our previous publication (Westrom et al.) was innovative, as it was the first to evaluate an anti-CD146 antibody, OI-3, as a carrier for the targeted delivery of beta-emitting ^177^Lu as a payload for potential anti-tumor effects in an osteosarcoma xenograft model^14^. In the present study, we further characterized murine and chimeric variants of the OI-3 antibody and evaluated its potential as a carrier for alpha-emitting RIT in CD146-positive tumors. In MM patients, immunohistochemistry (IHC) analysis has revealed more than 80% CD146 positive MM cells, whereas the CD146 signal is nearly absent in the healthy mesothelium^10^. CD146 expression has been observed in both epithelioid and sarcomatoid MM tumors^29,30^.

Lead-212 (^212^Pb), with a half-life of 10.6 h, is a beta-emitting isotope that can be effectively harnessed for targeted alpha therapy (TAT) because of its alpha-emitting offsprings, ^212^Bi and ^212^Po with half-lives of 61 min and 0.3 µs, respectively. The use of various bifunctional chelators allows for the attachment of ^212^Pb to specific targeting agents^31,32^. For ^212^Pb, S-2-(4-isothiocyanatobenzyl)-1,4,7,10-tetraaza-1,4,7,10-tetra(2-carbamoylmethyl) cyclododecane (p-SCN-Bn-TCMC, TCMC) is commonly used^33^. Moreover, ^212^Pb can be sourced from generators with longer half-lives, e.g. ^228^Th (1.9 years) or ^224^Ra (3.6 days), making it viable for large-scale production^34^.

Precise delivery of alpha-emitting isotopes to cancerous cells in body cavities is crucial for maximizing tumor destruction while minimizing harm to healthy tissues or negatively affecting cells of the immune system because of the short range of alpha particles (< 100 µm). Furthermore, TAT can induce a bystander effect, affecting neighboring cancer cells through non-targeted cytotoxic effects, greatly augmenting the effects of TAT^35^. Additionally, the abscopal effect, as demonstrated in the case of cutaneous squamous cell carcinoma treated with diffusing alpha emitter radiation therapy, suggests that alpha particle treatment can stimulate an immune-mediated response leading to tumor regression at sites distant from the irradiated area^36^. This phenomenon underscores the potential of alpha-emitting isotopes not only in directly targeted tumor destruction, but also in harnessing the body’s immune system for systemic anti-tumor effects. Moreover, the bystander effect, as observed in the dose-dependent growth delay of breast cancer xenografts treated with ^223^Ra, highlights the role of non-irradiated cells in contributing to the therapeutic outcome, indicating that the biological effects of alpha therapy extend beyond the physical range of alpha particles^37^. These insights into the bystander and abscopal effects enrich our understanding of the complex interplay between targeted alpha therapy, the tumor microenvironment, and the immune system, offering new ways for enhancing the efficacy of radiotherapy in cancer treatment. Furthermore, internalization of the ^212^Pb immunoconjugate into cancer cells was found to lessen the damage to neighboring healthy tissue and was suggested to have an advantage over non-internalizing RIC in destroying certain small tumors^38^. Clinical trials of radioimmunotherapy using intraperitoneally administered ^212^Pb-TCMC-trastuzumab (NCT01384253) have been conducted for ovarian cancer, where it showed potential anticancer effects and was found to be safe, with only mild and transient adverse events reported^39^. Recent preclinical studies have reported various ^212^Pb conjugates, including those targeting HER1^40–42^, B7-H3^43,44^, CSPG4^45^, CD37^46^, CD38^47^, and PSMA^48–51^. Based on this and the data presented herein, we suggest that coupling OI-3 with ^212^Pb could enhance the specificity of the antibody to alpha radiation cytotoxicity, thus offering a novel RIC.

Our key contributions include the confirmation of CD146 as a viable target for malignant peritoneal mesothelioma (MPM), preclinical evaluation of ^212^Pb-TCMC-OI-3, and demonstration of its potential in terms of affinity, selectivity, internalization, and therapeutic efficacy. These findings not only advance our understanding of targeted radionuclide therapy in MPM but also lay the groundwork for future clinical applications in other types of solid tumors.

## Results

### Cell surface expression of CD146 on mesothelioma cell lines and affinity measurements

**Fig. 1:**
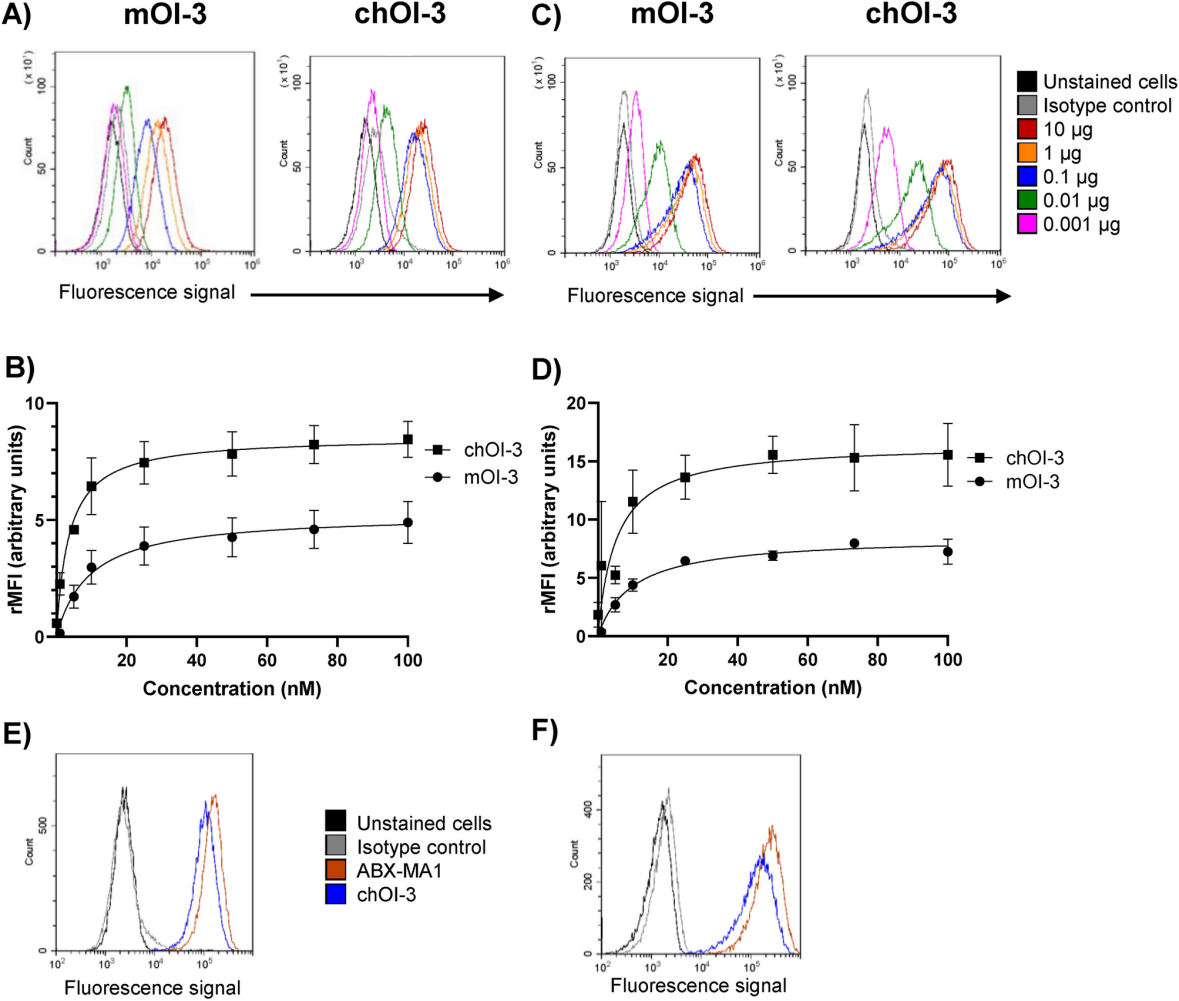
mOI-3, chOI-3 and ABX-MA1 bind to CD146 expressed on human mesothelioma cancer cell lines. (**A**) and (**C**) Representative flow cytometry histograms comparing the binding of increasing concentrations, 0.001–10 µg of mOI-3 Alexa 488 and chOI-3 Alexa 488 to (**A**) MSTO-211H, and (**C**) NCI-H226 cells for 30 min at 4 °C. Unstained cells, mouse, and human isotype controls were used as negative controls. (**B**) and (**D**) relative median fluorescence intensity (rMFI) for all samples was calculated based on rMFI of isotype control cells for MSTO-211H and NCI-H226, respectively. A one-site binding model (Y = B_max_ × X / (KD + X)) was used to analyze the data plotted as a hyperbolic curve (GraphPad Prism, La Jolla, USA). This analysis aimed to determine the apparent equilibrium dissociation constant (K_D_^APP^) specific to cancer cell lines. For MSTO-211H, K_D_^APP^ was 3.3 ± 0.3 nM and 9.2 ± 1.4 nM for chOI-3 and mOI-3 respectively. For NCI-H226, K_D_^APP^ was 5.9 ± 1.3 nM and 11.4 ± 4.0 nM for chOI-3 and mOI-3 respectively. (**E**) MSTO-211H, and (**F**) NCI-H226 cells were stained with human unlabeled antibody ABX-MA1 and chOI-3 for 30 min at 4 °C. Following the incubation, goat anti-human Alexa 488 antibody was used for 30 min 4 °C. In all experiments, only MitoTracker Red CMXRos and DRAQ5 double positive cells were included in the analysis. Samples were collected using CytoFlex. Data are representative of at least two or three independent experiments.

Flow cytometry analysis was performed to examine the cell surface expression of CD146 in the MSTO-211H and NCI-H226 human mesothelioma cancer cell lines. Specific binding of the mOI-3, chOI-3 and ABX-MA1 antibodies was observed (Fig. 1A,C,E,F), with isotype controls showing low binding (Fig. S1). Binding saturation was achieved at approximately 1–10 µg per 10^6^ cells for all antibodies and cell lines studied. The histogram peaks for MSTO-211H cells displayed a well-defined narrow shape, indicating a more homogeneous expression of CD146 compared to that in NCI-H226 cells (Fig. 1A,C). In contrast, the histogram peaks for NCI-H226 cells appeared broader, suggesting a more heterogeneous (varied) expression of CD146 (Fig. 1C). A saturation binding experiment was performed with decreasing antibody concentrations to measure antibody binding at equilibrium to a cell-surface antigen in the two cell lines. The goal was to determine the apparent K_D_^APP^ value for each cell line. The chOI-3 antibody showed a higher apparent affinity for binding to CD146 than the murine antibody based on rMFI data for both MSTO-211H (Fig. 1B) and NCI-H226 cells (Fig. 1D), with a 2.8- and 1.9-fold decrease in the K_D_^APP^ value, respectively. This was further evaluated by SPR analysis of human CD146. A 2.8-fold increase in the affinity of chOI-3 compared to that of the mOI-3 antibody was observed (Table 1). This finding suggests that the chimeric antibody may have an advantage in the binding profile to CD146 in these cells.

**Table 1:** Affinity measurement of mAbs. K_D_ of TCMC- and non-TCMC-conjugated mAbs by surface plasmon resonance (SPR) analysis with human CD146 antigen.

Antibody	Molar ratio of TCMC to mAb in conjugation reaction	K_D_ value by SPR with non-radiolabeled mAb (nM)
mOI-3	NA	5.1
chOI-3	NA	1.8
TCMC-chOI-3	5x	4.3
	10x	6.4
	15x	15.1
	20x	13.9

K_D_, the equilibrium dissociation constant.

The comparison of ABX-MA1 binding demonstrated a 1.4-fold higher binding of ABX-MA1 to MSTO-211H compared to that of chOI-3, and a 1.7-fold higher binding to NCI-H226 cells (Fig. 1E,F).

**Fig. 2:**
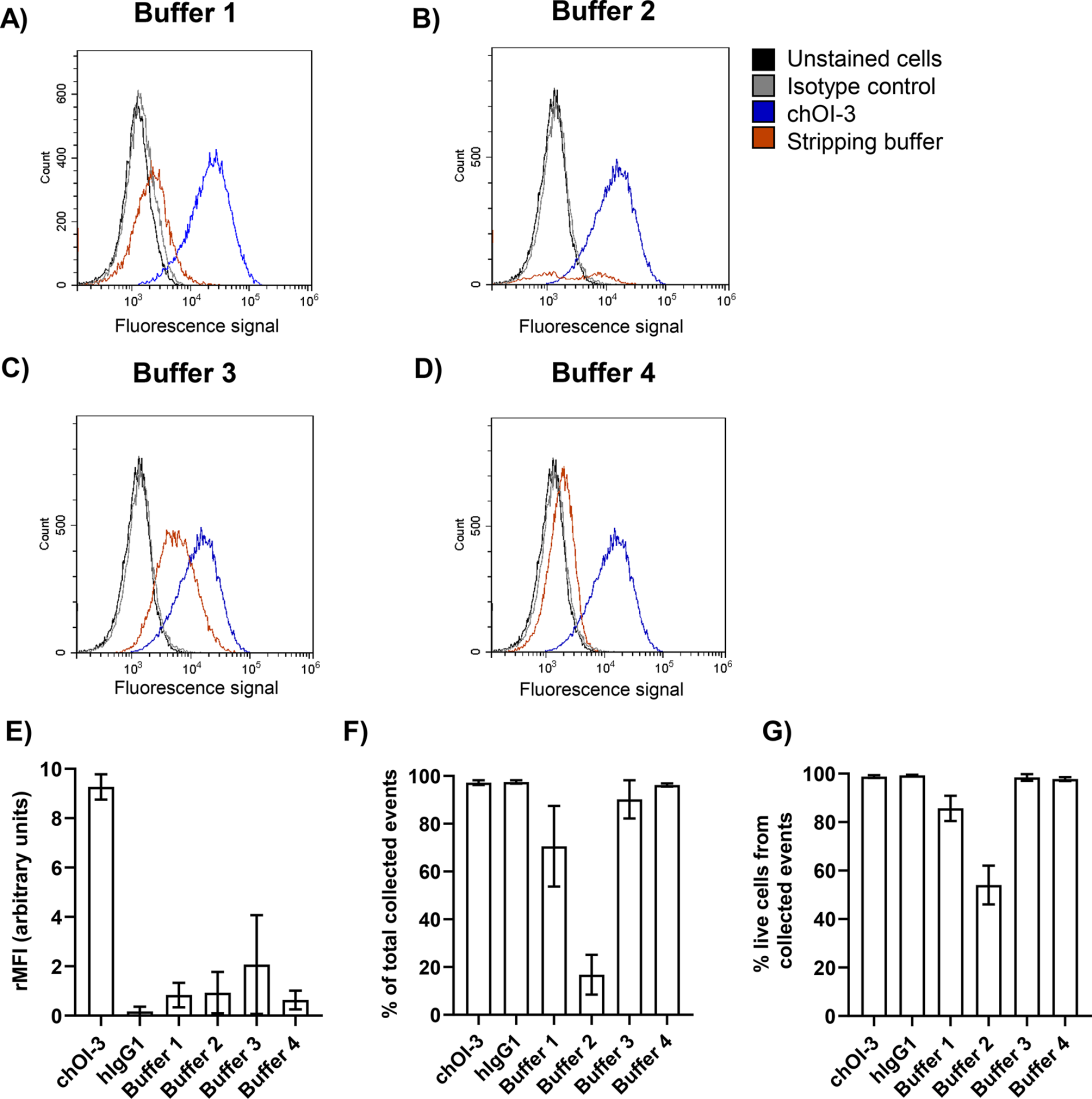
Buffer 4 (trypsin digestion) was the most efficient method for removing the chOI-3-CD146 complex from the cell surface of the mesothelioma cell line NCI-H226 compared to other buffers. (**A**–**D**) NCI-H226 cells were incubated with 0.25 µg chOI-3 Alexa 488 for 30 min at 4 °C. After two washes with PBS, cells were kept in flow buffer (untreated samples) or in stripping buffers for indicated periods of time at 37 °C in an incubator: (**A**) 50 mM glycine, 150 nM NaCl, pH 2.6 for 10 min; (**B**) 1% citric acid, 0.5 M NaCl, pH 3 for 45 min; (**C**) 1% w/v pepsin (diluted 1:100) in PBS pH 3–4 for 45 min; and (**D**) 0.01 g/mL trypsin in PBS pH 9 for 60 min. The cells were then washed twice with the flow buffer. Only MitoTracker Red CMXRos and DRAQ5 double positive cells were included in analysis. Samples were immediately collected using CytoFlex. Unstained cells and isotype control stained cells served as a negative control and chOI-3 Alexa 488 stained cells as positive controls. (**E**) The rMFI for all samples was calculated based on the MFI of the unstained cells. Each point was calculated as the mean of triplicate rMFI with the SD for each experiment. Data are presented as mean ± SD from at least three independent experiments. (**F**) The percentage was calculated as the mean of triplicate percentages of collected events from the expected total number of events with SD for each experiment. Data are presented as mean ± SD from at least three independent experiments. (**G**) Viability was measured as the mean percentage of MitoTracker Red CMXRos and DRAQ5 double-positive cells with SD from triplicate samples collected after incubation with each buffer. Data are presented as mean ± SD from at least three independent experiments.

Binding and internalization of chOI-3 Alexa 488 were investigated in NCI-H226 cells (Fig. 2). To optimize the stripping conditions, four different buffers were tested to determine the most efficient method for removing surface-bound chOI-3 Alexa 488, while ensuring that the stripping process did not lyse the cells (Fig. 2, Table 2). Buffer 1 (50 mM glycine, 150 nM NaCl pH 2.6) treatment for 10 min led to 91% loss of the signal with 81.2 ± 10.6% viability (Fig. 2A,E,F,G and Table 2). Cells treated with Buffer 2 (1% citric acid, 0.5 M NaCl, pH 3) for 45 min lost 90% Alexa 488 signal, with 58.0 ± 7.1% viable cells (Fig. 2B). An increase in the citric acid concentration led to increased cell death (data not shown). The use of buffer 3 (1% w/v pepsin (diluted 1:100) in PBS pH 3–4) for 45 min resulted in 98% signal loss, while the viability was 95.8 ± 5.6 (Fig. 2C and Table 2). Treatment with buffer 4 (0.01 g/mL trypsin in PBS, pH 9) for 60 min led to a 93% loss of signal with a viability of 97.8 ± 0.8 (Fig. 2D and Table 2). Therefore, buffer 4 was selected as the most efficient cell surface stripping buffer with the least effect on cell viability.

**Table 2:** Optimization of stripping buffers. NCI-H226 cells were incubated with 0.25 µg chOI-3 Alexa 488 for 30 min. The cells were either left untreated (total signal) or exposed to stripping buffers to remove the surface-bound antibodies (internalized signal). Stripping buffers included 1) 50 mM glycine, 150 nM NaCl, pH 2.6 for 10 min; 2) 1% citric acid, 0.5 M NaCl, pH 3 for 45 min; 3) 1% w/v pepsin (diluted 1:100) in PBS pH 3–4 for 45 min; and 4) 0.01 g/mL trypsin in PBS pH 9 for 60 min. For each buffer, the percentage of collected events out of the total events was displayed. Only live cells were included in this analysis, and the percentage of viable cells was calculated from the total collected cells, as shown in the table. The relative fluorescence intensity (rMFI) of each sample was measured using viable cells. Data are representative of at least two to four independent experiments.

	Non-treated cells	hIgG	Buffer 1	Buffer 2	Buffer 3	Buffer 4
rMFI	9.3 ± 0.5	0.3 ± 0 .1	0.8 ± 0.5	0.9 ± 0.8	2.1 ± 2.0	0.6 ± 0.4
% of total events	97.6 ± 0.7	97.7 ± 0.7	70.6 ± 16.9	16.8 ± 8.3	86.9 ± 13.6	96.2 ± 0.7
% viable cells	98.9 ± 0.5	99.1 ± 0.5	81.2 ± 10.6	58.0 ± 7.1	95.8 ± 5.6	97.8 ± 0.8

### Cellular internalization kinetics of surface bound chOI-3 and mOI-3

**Fig. 3:**
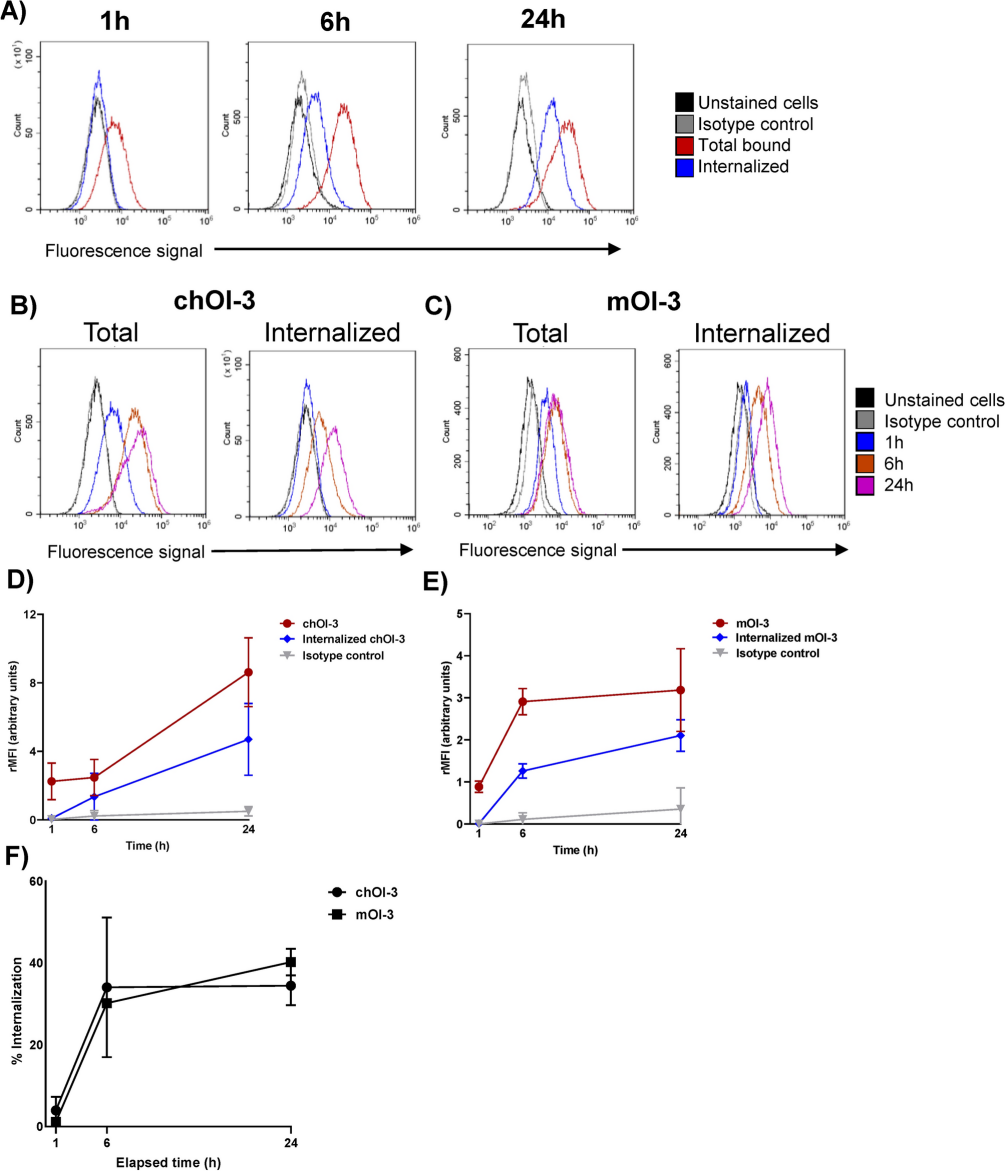
Measurement of chOI-3 and mOI-3 Alexa 488 antibody total binding and internalization compared to hIgG1 Alexa 488 in MSTO-211H during 24-h time period. (**A**) MSTO-211H cells were incubated with 0.25 µg ChOI-3 Alexa 488 and placed in 37 °C incubator with shaking in cell media (RPMI 1640 with 10% fetal bovine serum and 1% penicillin–streptomycin). The cells were collected at 1, 6 or 24 h and after two washes with PBS, cells were kept in 0.01 g/mL trypsin (stripping buffer 4), for 1 h at 37 °C, or left untreated. Only MitoTracker Red CMXRos and DRAQ5 double positive cells were included in analysis. (**B**) and (**C**) Representative histograms of trypsin-stripped MSTO-211H cells on the right and total signal on the left for samples at 1 h, 6 h, and 24 h for chOI-3 and mOI-3, respectively. (**D**) and (**E**) The relative median fluorescence intensity (rMFI) for triplicate samples was calculated based on the MFI of unstained cells for each experiment. Each point represents the mean of triplicate rMFI values with SD. The data represent the mean ± SD of four independent experiments.

**Fig. 4:**
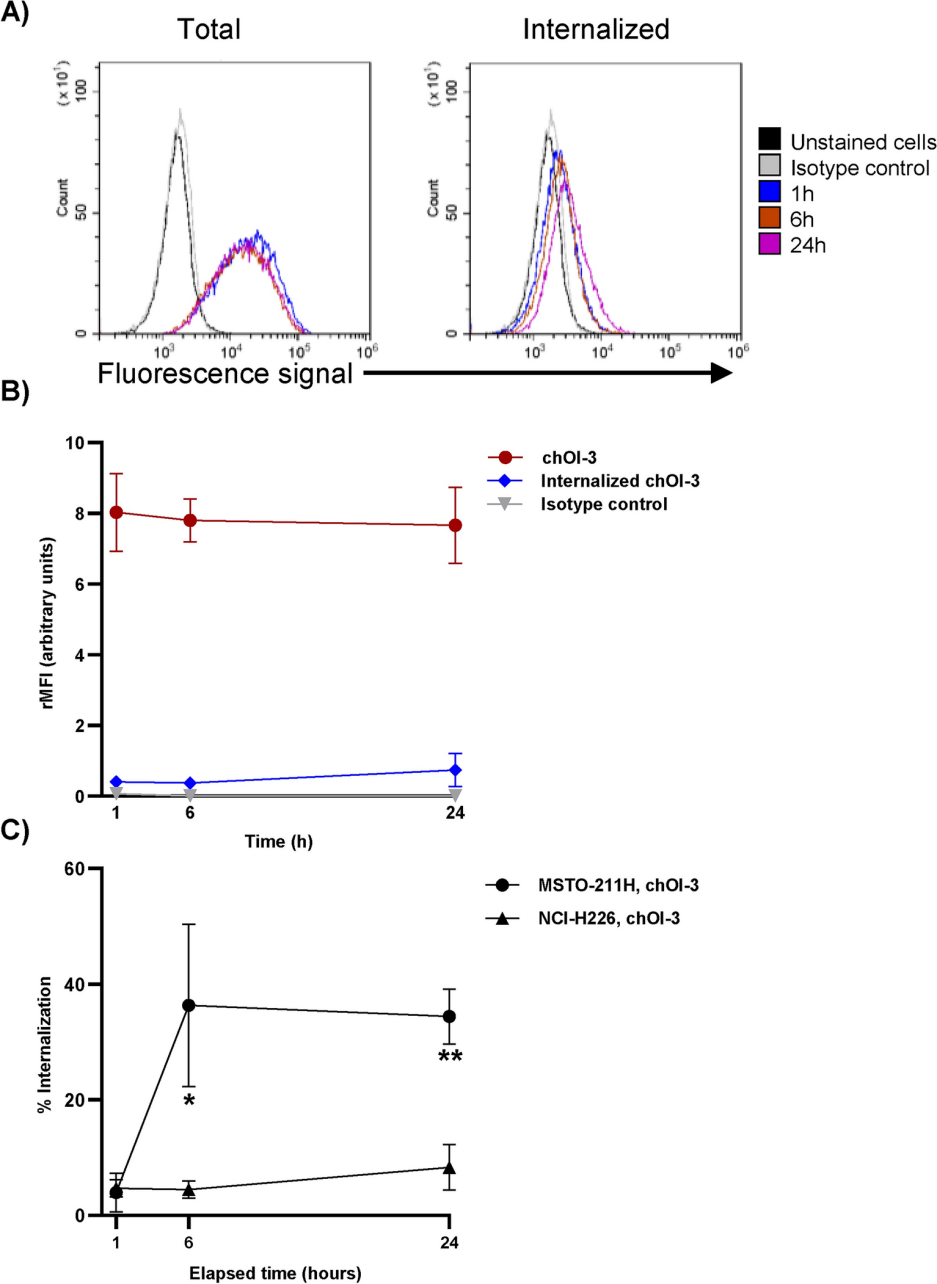
The measurement of total binding and internalization of the chOI-3 Alexa 488 antibody was compared to that of hIgG1 Alexa 488 in NCI-H226 during 24-h time period. (**A**) NCI-H226 cells were incubated with 0.25 µg chOI-3 Alexa 488 for 1, 6 or 24 h at 37 °C in incubator with shaking in cell media (RPMI 1640 with 10% fetal bovine serum and 1% penicillin–streptomycin). Cells were then either left untreated to measure total bound antibodies via Alexa 488 signal (on the left) or exposed to trypsin (buffer 4) to remove surface-bound antibodies to measure internalized antibodies via Alexa 488 signal (on the right). All samples were analyzed in triplicate. Only MitoTracker Red CMXRos and DRAQ5 double positive cells were included in analysis. (**B**) The relative median fluorescence intensity (rMFI) for triplicate samples was calculated based on the MFI of unstained cells for each experiment. Each point represents the mean of triplicate rMFI with SD. The data represent the mean ± SD of three independent experiments. (**C**) The percentage of internalized signals was calculated in MSTO-211H and NCI-H226 cells incubated with chOI-3. P-values were calculated using an unpaired t-test with correction for multiple comparisons using the Holm-Sidak method; *p_adj_ < 0.01 and **p_adj_ < 0.0006.

In some small-volume xenograft tumors, it has been reported that RIC internalization may enhance tumor cell killing *in vivo* compared to non-internalizing antibodies and reduce the damage to neighboring healthy tissues^38^. Therefore, the internalization of mOI-3 in MSTO-211H cells and chOI-3 in MSTO-211H and NCI-H226 cells was investigated (Figs. 3 and 4). After 1 h of chOI-3 Alexa 488 incubation with MSTO-211H cells followed by stripping of surface mAb with trypsin treatment, the Alexa 488 signal was 5.3 ± 2.6% of the total signal detected in the non-stripped cells (Fig. 3A and Table 3, left column). At 6 h and 24 h, the signal was 36.3 ± 14.0% and 34.4 ± 5.4%, respectively, suggesting an increase in chOI-3 internalization over a 6-h time period beyond which the rate reaches a plateau, indicating a stabilization of the signal (Fig. 3 and Table 3). In addition, an increase in total rMFI (cell surface and internalized signal) and internalized rMFI was observed during the incubation period (Fig. 3D). Thus, MSTO-211H cells may serve as a good candidate to examine ^212^Pb-chOI-3 binding efficiency followed by RIC internalization.

**Table 3:** Percent internalization of Alexa 488 mAb in MSTO-211H and NCI-H226. MSTO-211H and NCI-H226 cells were incubated with 0.25 µg OI-3 Alexa 488 (mOI-3 or chOI-3) for 1, 6 or 24 h. The cells were either left untreated (total signal) or exposed to trypsin (buffer 4) to remove the surface-bound antibodies (internalized signal). Only live cells were included in this analysis. The relative fluorescence intensity (rMFI) of each sample was measured, and the percentage of internalized signal was calculated as the internalized rMFI signal (trypsin-stripped cells) compared to the total-bound rMFI (non-stripped cells). Data are representative of at least two to four independent experiments.

Incubation time (h)	MSTO-211H cells		NCI-H226 cells
	mOI-3 (%)	chOI-3 (%)	chOI-3 (%)
1	1.1 ± 0.2	5.3 ± 2.6	4.7 ± 1.5
6	30.2 ± 0.6	36.3 ± 14.0	4.5 ± 1.5
24	40.2 ± 3.2	34.4 ± 5.4	8.3 ± 3.9

mOI-3 was examined for internalization into MSTO-211H cells. Using the same experimental setup, an increase in the Alexa 488 signal in the cells was observed over a 24-h time period (Fig. 3C). When cells were stripped, at 1 h, the internalized signal was 1.1 ± 0.2%, while at 6 h it increased to 30.2 ± 0.6%, and at 24 h it reached 40.2 ± 3.2% of the total signal (Fig. 3E and Table 3, middle). No statistically significant differences were found when the percentages of internalized mOI-3 and chOI-3 signals were compared (Fig. 3F and Table 3), although the mOI-3 rMFI was consistently lower than the chOI-3 rMFI in the MSTO-211H cells (Fig. 3D,E).

NCI-H226 cells displayed different dynamics of chOI-3 binding and internalization compared to MSTO-211H cells (Fig. 4B). Trypsin treatment for 1 h resulted in a 4.7 ± 1.5% internalized signal (Fig. 4B and Table 3, left), suggesting negligible antibody internalization (Fig. 4B). This lack of internalization persisted throughout the 24-h incubation with 4.5 ± 1.5%, and 8.3 ± 3.9% internalized signal at 6 h and 24 h respectively (Fig. 4B and Table 3). When total rMFI was calculated, the value did not change over the 24-h time period (Fig. 4B).

There was a statistically significant difference in internalized chOI-3 levels between NCI-H226 and MSTO-211H cells at 6- and 24-h time points (Fig. 4C). This suggests that NCI-H226 cells may not be a good candidate for studying the internalization of ^212^Pb-TCMC-OI-3.

**Fig. 5:**
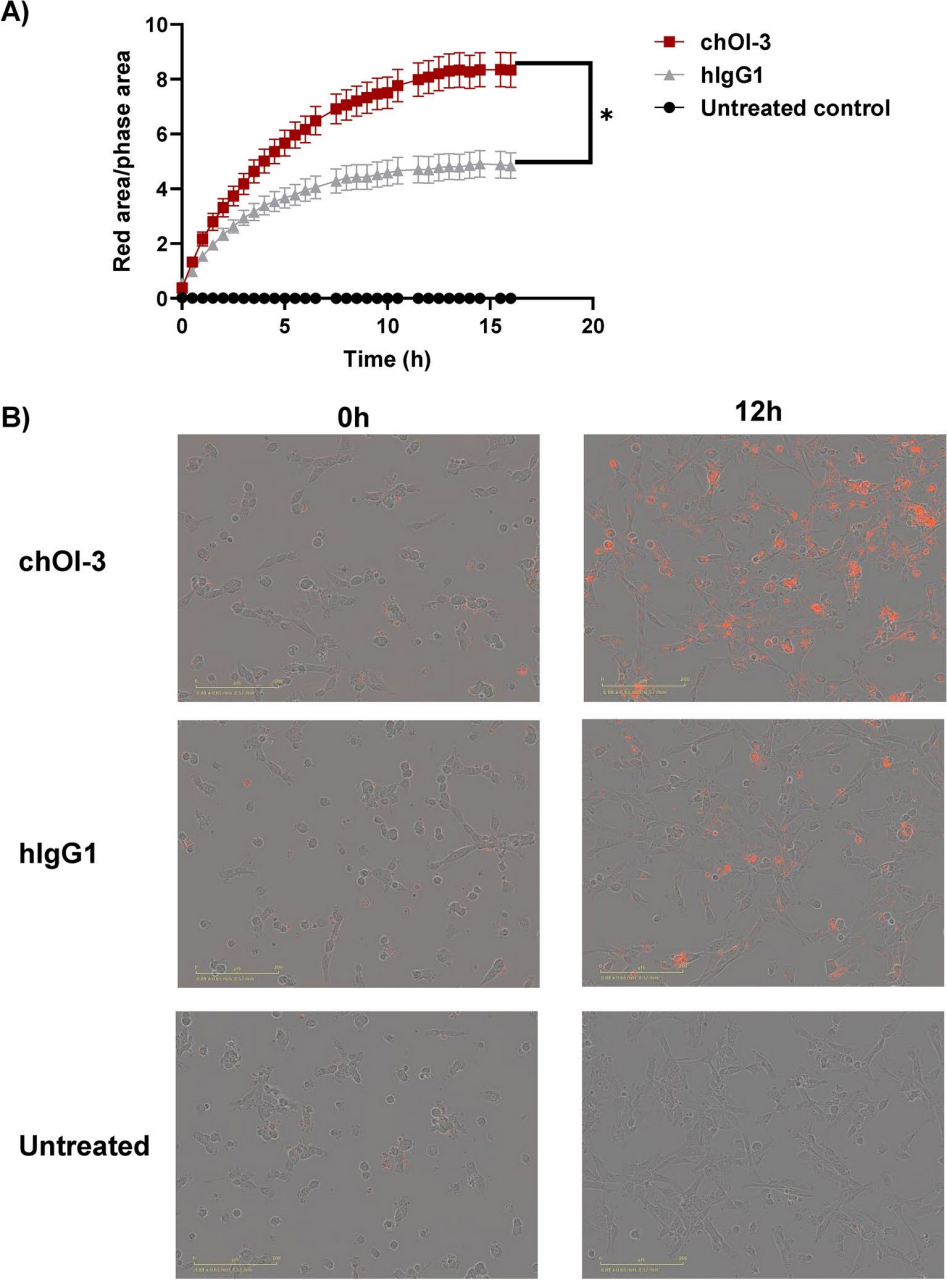
Real-time Monitoring of chOI-3 and Isotype Control Antibody Internalization in MSTO-211H cells. (**A**) MSTO-211H cells (1 × 10^3^ cells/well) were seeded in 96-well plates. After 24 h, the medium was removed, and the cells were incubated with either IncuCyte FabFluor-pH red-labeled chOI-3 antibody or human IgG1 isotype control (0.25 μg/mL) for 16 h. Live-cell imaging was performed every 30 min using an Incucyte Live-Cell imaging system (S3, Sartorius, Göttingen, Germany) to capture phase contrast and red fluorescence images (10 × magnification). Cellular internalization of the antibodies was measured over time using the fluorescence signal generated by IncuCyte FabFluor-pH Red reagent. The mean area under the curve (AUC) was calculated for each treatment group and compared using unpaired t-tests. Data are presented as the mean AUC ± standard error of the mean (SEM) from two independent experiments. *p < 0.0002 for chOI-3 compared with hIgG. (**B**) Representative images were obtained before and 12 h after the start of the experiment. Images represent signals in untreated, chOI-3 and hIgG1 treated samples.

The IncuCyte live-cell imaging system automatically captures images and measures antibody internalization over extended periods of time and does not require cell manipulation. The internalization kinetics of chOI-3 and hlgG in MSTO-211H cells were monitored using IncuCyte for 16 h (Fig. 5A). Representative images of antibody internalization at 0 h and 12 h are shown (Fig. 5B).

### Species cross reactivity evaluation by immunohistochemistry

Cross-reactivity between species happens when an antibody identifies antigens that are present in different species. These antigens may display similar three-dimensional structural regions known as epitopes, which allows the antibody for one antigen to recognize antigens in different species^52^. The cross-reactivity of antibodies plays an important role during the drug development and approval process. Antibody species cross reactivity was evaluated in selected normal frozen tissue microarray sections from mouse, rat, and human. To avoid issues with background staining, mOI-3 was used on human slides, whereas chOI-3 was used on mouse and rat slides. The data in Table 4 indicate a positive staining pattern and overlap in the selected tissues for CD146 in all the three species. The H scoring system mostly indicated + 1 and + 2, suggesting a weak-to-intermediate signal. Therefore, species cross-reactivity to rodent CD146 indicates that rodent models can be used in preclinical safety studies. Human and mouse CD146 proteins have 76.2% sequence identity, whereas human and rat share 74% sequence identity at the amino acid level (Fig. S2).

**Table 4:** CD146 expression in selected normal tissues from mouse, rat, and human. The different tissues selected for species cross-reactivity with mOI-3 and chOI-3 scoring system H (0 to + 3).

Antibody	Tissue	Heart	Liver	Pancreas	Stomach and small intestine	Spleen
mOI-3	Human	1 + : CM 2 + : EC	1 + : EC	1 + : GEC	1 + : GEC	1 + : LC
chOI-3	Mouse	1 + : CM	1 + : HC	1 + : GEC	1 + : GEC	1 + : LC
chOI-3	Rat	1 + : CM	1 + : HC	1 + : GEC	1 + : GEC	3 + : LC
Cell type		Cardiac myocytes (CM) Endothelial cells (EC)	Endothelial cells (EC) Hepatocytes (HC)	Glandular epithelial cells (GEC)		Lymphocytes (LC)

### Immunoreactive fraction (IRF)

A 5–20-fold molar excess of TCMC is often used for antibody conjugation with ^212^Pb *in* *in vitro* and *in vivo* studies^53^. chOI-3 was conjugated at different molar ratios of TCMC (5-, 10-, 15-, and 20-fold). IRF assay on the OHS cell line was performed using ^212^Pb-labeled TCMC-mOI-3 and TCMC-chOI-3 at different molar ratios of TCMC. The radiochemical purity was > 96% for all ^212^Pb-TCMC-mAbs. A decrease in IRF was observed as the TCMC molar ratio increased to its highest value (Table 5). An increase in the TCMC molar ratio may negatively affect the affinity of the TCMC-conjugated chimeric antibody, as supported by the K_D_-values obtained from SPR (Table 1). The optimal ratio of TCMC to antibodies was determined to be 5:1. Hence, a 5:1 TCMC-to-antibody ratio was used for the *in vivo* studies.

**Table 5:** Immunoreactive affinity of ^212^Pb-TCMC-mAbs. The average radiochemical purity (RCP) was > 96% for all ^212^Pb-TCMC-mAbs, and the average immunoreactive fraction (IRF) was expressed as the cell bound percentage of ^212^Pb-TCMC-mAb ± SD.

Antibody	Molar ratio of TCMC to mAb in chelation reaction	Average RCP of ^212^Pb-TCMC-mAb (%) ± SD	Average IRF (%) ± SD of ^212^Pb-TCMC-mAb
TCMC-mOI-3	5x	99.2 ± 1.0 (n = 26)	59.2 ± 7.1 (n = 26)
TCMC-chOI-3	5x	98.8 ± 1.0 (n = 12)	63.0 ± 3.9 (n = 12)
	10x	98.9 ± 0.9 (n = 11)	51.5 ± 7.8 (n = 11)
	15x	98.6 ± 1.7 (n = 5)	39.6 ± 7.1 (n = 5)
	20x	99.7 ± 0.6 (n = 3)	33.3 ± 1.2 (n = 3)

SD, standard deviation; n, number of samples.

### Internalization of ^212^Pb-TCMC-chOI-3 in MSTO-211H cells

**Fig. 6:**
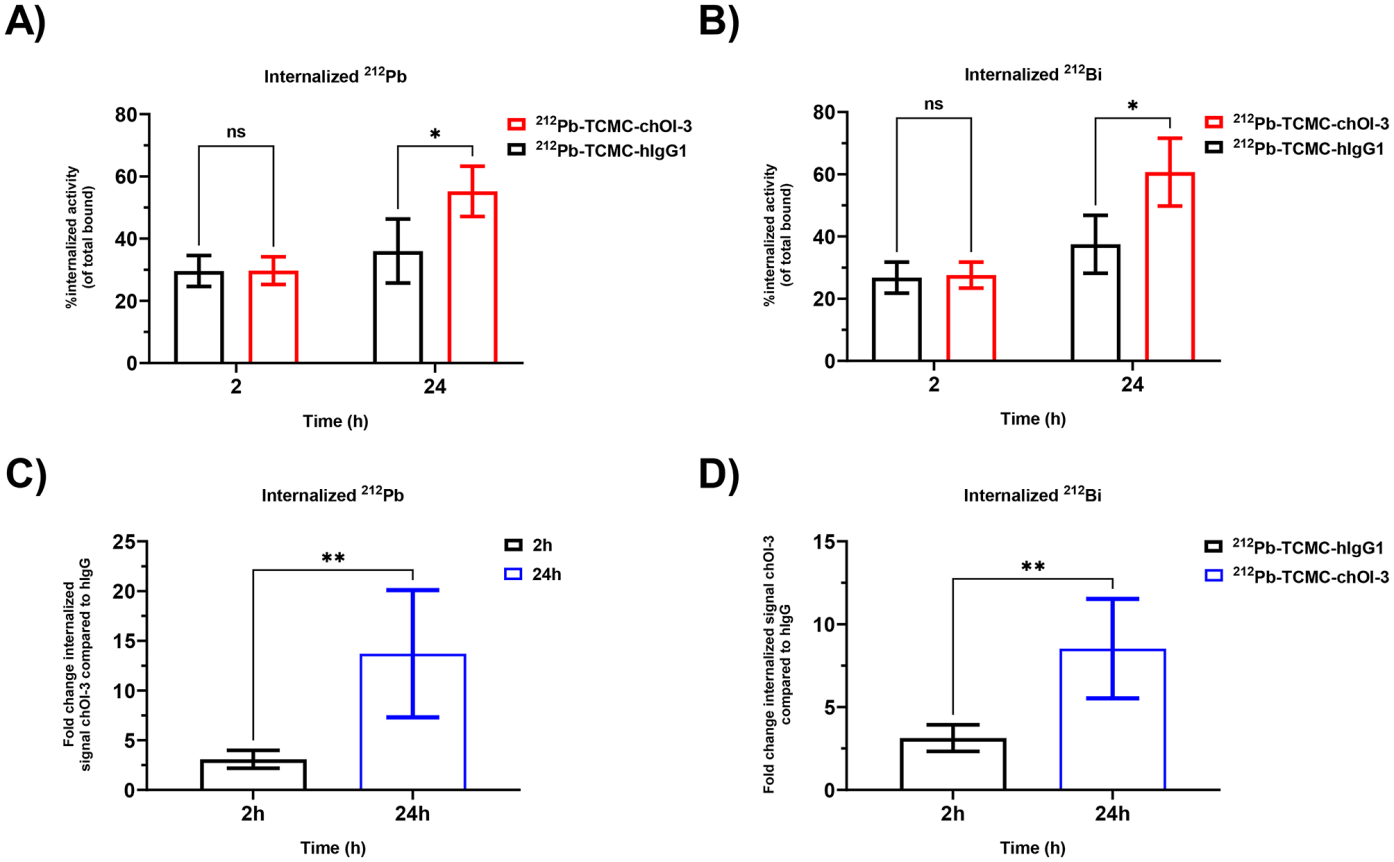
Internalization of ^212^Pb and ^212^Bi atoms in MSTO-211H cells. The cells were incubated with 5–10 kBq/ml ^212^Pb-TCMC-chOI-3 or 5–10 kBq/ml ^212^Pb-TCMC-hIgG1 for 2 and 24 h in a 37 °C incubator. Following incubation, the cells were treated with buffer 4 for 1 h at 37 °C to remove the surface-bound ^212^Pb-TCMC-mAbs. The soluble fraction (cell-surface bound) and cell pellet (internalized) were collected after three washes with the flow buffer. Cell surface-bound and internalized activities were measured using Hidex gamma counter. The total number of atoms was calculated by adding surface bound and internalized values. The percentage of internalized atoms per cell was calculated as the internalized signal/total signal. The fold change was calculated as the number of ^212^Pb or ^212^Bi atoms internalized by chOI-3 divided by internalized atoms hIgG, i.e. atom_internalized chOI-3_/atom_internalized hIgG_. (**A**) %internalized ^212^Pb atoms per cell and (**B**) %internalized ^212^Bi atoms per cell. (**C**) and (**D**) indicate the fold change of the internalized signal chOI-3 compared to hIgG at 2 h and 24 h for ^212^Pb and ^212^Bi atoms, respectively. The results are presented as the average of five independent experiments, with error bars. p-values were calculated using an unpaired t-test with correction for multiple comparisons using the Holm-Sidak method (*p_adj_ < 0.01, **p_adj_ < 0.006).

The internalization kinetics of tenfold excess TCMC ^212^Pb-labeled chOI-3 (^212^Pb-TCMC-chOI-3) were examined and compared with those of the human isotype control, ^212^Pb-TCMC-hIgG1 (tenfold TCMC excess), in MSTO-211H cells. Kinetic studies of the ^212^Pb-TCMC-chOI-3 antibody were performed because of its higher affinity for the antigen than the murine version (Table 1). This study evaluated the dynamics of antibody internalization at 2 h and 24 h. A significant increase in internalized ^212^Pb and ^212^Bi atoms per cell was observed with chOI-3 compared with hIgG1 at 24 h (*p* < 0.01). At 2 h and 24 h, there was a 3.1- and 13.7-fold increase in internalized ^212^Pb atoms per cell and 3.1- and 8.5-fold increase in internalized ^212^Bi atoms per cell at 2 h and 24 h, respectively (Fig. 6A, B, Table 6) with a significant (*p* < 0.006) increase from 2 to 24 h for both ^212^Pb and ^212^Bi (Fig. 6C,D). This notion supports the observed increase in the internalized signal of chOI-3 in MSTO-211H cells in both flow cytometry and IncuCyte. These results indicate the internalization of ^212^Pb and, more importantly, ^212^Bi in MSTO-211H cells.

**Table 6:** ^212^Pb and ^212^Bi internalized atoms and fold change for MSTO-211H cell line at 2 and 24 h. The data represent the internalized ^212^Pb and ^212^Bi atoms per cell from ^212^Pb-TCMC-chOI-3 and ^212^Pb-TCMC-hIgG1 from five independent experiments. The percentage of internalized atoms per cell was calculated as the internalized signal/total signal. The fold change was calculated as the number of ^212^Pb or ^212^Bi atoms internalized by chOI-3 divided by internalized atoms hIgG, i.e. atom_internalized chOI-3_/atom_internalized hIgG_.

Sample, timepoint		Average ± SD of ^212^Pb internalized atoms per cell		Average ± SD of ^212^Bi internalized atoms per cell	
		%Internalized	Fold change	%Internalized	Fold change
^212^Pb-TCMC-hIgG1	2 h	29.6 ± 5.0%	NA	26.8 ± 5.0%	NA
	24 h	36.0 ± 10.3%	NA	37.5 ± 9.3%	NA
^212^Pb-TCMC-chOI-3	2 h	29.7 ± 4.5%	3.1 ± 0.9	27.6 ± 4.2%	3.1 ± 0.8
	24 h	55.2 ± 8.1%	13.7 ± 6.4	60.7 ± 10.9%	8.5 ± 3.0

SD, standard deviation. NA, not applicable.

### Therapeutic efficacy of ^212^Pb-TCMC-mOI-3 in an intraperitoneal xenograft model of mesothelioma

**Fig. 7:**
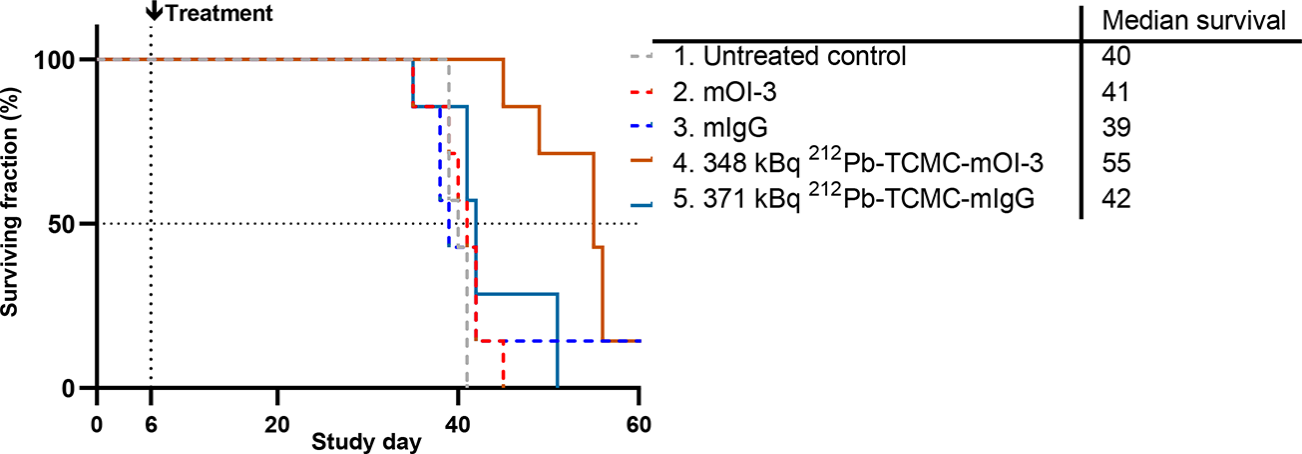
Anti-tumor effect of ^212^Pb-TCMC-mOI-3. Kaplan–Meier curves of nude mice inoculated intraperitoneally with MSTO-211H cells on day zero and treated intraperitoneally on day 6 with 1. Saline, 2. mOI-3, and 3. mIgG, and 4. 348 kBq ^212^Pb-TCMC-mOI-3 and 5. 371 kBq ^212^Pb-TCMC-mIgG. mAb amount was 10 µg. Seven mice were used for each group. The median survival (days) was calculated for all treatment groups. There were censored mice on day 165 (one mouse in group 3 and 4), corresponding to three times the median survival time of the longest surviving group at which the study was terminated.

Therapeutic efficacy was assessed in mice with i.p. mesothelioma xenograft MSTO-211H. Mice treated with ^212^Pb-TCMC-mOI-3 were compared to those treated with ^212^Pb-TCMC-mIgG, saline (control mice), unlabeled mOI-3, or mIgG (Fig. 7). During the course of the study, mice treated with ^212^Pb-TCMC-mOI-3 showed a significantly prolonged survival time compared with saline-treated mice (p_adj_ = 0.008) and non-labeled mOI-3 controls (p_adj_ = 0.01) (Fig. 7). The median survival time of the ^212^Pb-TCMC-mOI-3 group was 55 days, whereas the median survival times for saline, OI-3, and ^212^Pb-TCMC-mIgG groups were 40, 41, 39, and 42 days, respectively. The median survival of mice was 23–29% (13–16 days) longer in the ^212^Pb-labeled mOI-3 group than in the other groups (Fig. 7). The body weight changes in the mice are shown in Fig. S3. These results showed that there was no significant difference in the body weight of the mice between any of the groups (*p* > 0.05).

### Discussion

Current therapies for mesothelioma, including chemotherapy and immune checkpoint inhibitors (ICIs), offer limited survival benefits, as highlighted in recent studies^54,55^. Meirson et al. critically assess the effectiveness of frontline therapies, noting high costs and modest survival improvements with ICIs, as well as with combinations like cisplatin plus pemetrexed and the addition of bevacizumab^54^. Furthermore, Gray et al. discuss the uncertain clinical value of ICIs, emphasizing that real-world data suggest limited survival benefits, particularly for the epithelioid subtype^55^. These insights underscore the need for innovative therapies, where one approach could be CD146-targeting by intracavitary administration of ^212^Pb-TCMC-OI-3.

The current study supports the potential of targeting CD146 expressed in MPM using an alpha-emitting RIC. The expression of CD146 was confirmed in two human MM cell lines tested *in vitro*, MSTO-211H and NCI-H226, consistent with previous findings of CD146 expression in MM patient samples using IHC^56^. The K_D_ values obtained from the saturation binding assay by flow cytometry and SPR were within the range (3–11 nM) reported for other CD146-binding antibodies^14,16,17,23^, demonstrating a comparable affinity of OI-3 antibodies to others. In the present study, a 2.8-fold increase in antibody affinity was observed for the chimeric antibody compared to murine (Table 1). This finding aligns with previous research by Westrøm et al.^14^, where a Schatchard analysis on osteosarcoma cell lines revealed a 1.4-fold increase in affinity for chOI-3; specifically, the K_D_ were 1.8 ± 1.2 and 2.6 ± 0.3 nM for mOI-3 and chOI-3.1, respectively, indicating that the affinities on these two cell lines were similar.

It is a concern that approximately 30% of ^212^Bi, the longest-living daughter of ^212^Pb, may dissociate from TCMC after ^212^Pb decay, and the redistribution of the daughter nuclide *in vivo* may lead to damage to healthy tissues^57^. The internalization process may reduce the distribution of free ^212^Bi through the systemic circulation, thus increasing retention at the tumor site^33^. This notion is supported by studies conducted by Stenberg et al., who demonstrated enhanced *in vivo* tumor retention using an internalizing peptide radiolabeled with ^212^Pb^49,50^. Additionally, RIC internalization may play a beneficial role in augmenting tumor cell killing *in vivo* compared with non-internalizing antibodies in some small-volume xenograft tumors using radiolabeled trastuzumab^38^. CD146 is an attractive target for ^212^Pb-mAb because CD146-bound mAb is internalized by MSTO-H226 cells, as demonstrated using anti-CD146 antibody coated nanoparticles^56^ as well as in other CD146 expressing cancer cells (Nollet et al.^22^ and Hernandez et al.^23^).

Our study focused on the internalization dynamics of chOI-3 and mOI-3 antibodies in MSTO-211H and NCI-H226 mesothelioma cell lines, with particular interest in the chOI-3 variant because of its higher antigen affinity. In MSTO-211H cells, chOI-3 showed a progressive increase in internalization, reaching a plateau after 6 h, suggesting an efficient uptake mechanism. This was in contrast to the negligible internalization observed in NCI-H226 cells, indicating cell line-specific internalization capabilities. Comparatively, mOI-3 showed a consistent increase in internalization over 24 h in MSTO-211H cells, achieving higher levels than chOI-3, without significant differences in the rate of uptake between the two antibody variants (Figs. 3 and 4). The IncuCyte system’s capability to capture real-time images without requiring cell manipulation provided valuable insights, revealing a steady rise in chOI-3 internalization that stabilized at approximately 10 h with a statistically significant difference in internalization rate compared to the isotype control (Fig. 5).

The increased rate of ^212^Bi internalization over time suggests that once ^212^Bi is generated within the cell from the decay of internalized ^212^Pb-chOI-3, it may remain localized within the cancer cell. This process may facilitate the retention of alpha-emitting radionuclides directly at the tumor site, thereby potentially reducing radiation exposure to the surrounding healthy tissues. Future studies should explore the underlying mechanisms driving the differences in internalization rates between mesothelioma cell lines and the impact of radiolabeling on antibody behavior within the cellular environment.

The specificity of the OI-3 antibody for CD146 is essential for its therapeutic application in malignant mesothelioma. While CD146 is overexpressed in malignant cells, it is also present in normal endothelial and smooth muscle cells, albeit at lower levels^9^. Our immunohistochemistry studies (Table 4) show that OI-3 interacts with endothelial cells in multiple organs, with reactivity ranging from mostly + 1 to + 2 on a scale of 0 to + 3, to human, murine and rat CD146 supporting that studies in rodent models have high translational value related to normal tissue binding and biodistribution pattern. The biodistribution data with mOI-3 and chOI-3 from Westrom et al. demonstrates favorable tumor-to-normal tissue ratios and low accumulation in normal tissues at later time points (up to 14 days)^14^. It is important to note that the studies by Westrom et al. examined systemic (intravenous) administration of OI-3, where the potential for binding to normal endothelium is likely higher compared to the intracavitary approach evaluated herein.

With a half-life of 10.6 h, ^212^Pb allows sufficient time for the antibody to distribute within the peritoneal cavity and target tumor cells, while the subsequent emission of alpha particles by its decay product, ^212^Bi, ensures minimal damage to surrounding healthy tissues owing to its short path length^58^. This characteristic is crucial for effectively treating small tumor nodules and micrometastases with high precision. Furthermore, the localized delivery of ^212^Pb-labeled OI-3 directly into the peritoneal cavity achieves a concentrated radiopharmaceutical presence at the disease site. This approach ensures that most radioactive decay occurs within the peritoneal cavity, significantly mitigating the risk of systemic side effects commonly associated with intravenous RIT and leveraging the short half-life of ^212^Pb to enhance safety and efficacy^59^. The significant increase in survival time in mice treated with ^212^Pb-TCMC-mOI-3, with a median survival ratio of 1.3, coupled with the absence of significant differences in body weight among the experimental groups, supports the therapeutic efficacy of ^212^Pb-TCMC-mOI-3 treatment.

Comprehensive preclinical toxicity studies will be required to fully assess the safety profile of a selected OI-3 antibody variant before clinical translation. Studies will be needed to evaluate both short- and long-term toxicity at a range of doses, including histopathological and blood chemistry analyses, to assess potential off-target effects, particularly on vascular tissues expressing CD146. Although the localized administration approach may help mitigate systemic toxicity, this requires further validation. In conclusion, our findings provide evidence that ^212^Pb-TCMC-OI-3 effectively interacts with cell surface targets, leading to internalization *in vitro* and can improve survival in mice with intraperitoneal mesothelioma.

## Material and methods

### Preparation of antibodies

Murine OI-3 (mOI-3) and IgG1 chimeric variants of OI-3 (chOI-3) were developed as described previously^14,60^. The antibody-producing cell line CHO was propagated for separation and affinity purification of antibodies by Diatec Monoclonals (Oslo, Norway) for mOI-3. Pro TurboCHO cells were used for the production of chOI-3 by Genscript (Rijswijk, Netherlands). All antibodies were > 99% pure (SEC-HPLC) and endotoxin levels were ≤ 0.1 EU/mg as determined by the manufacturer. Anti-CD146 human mAb, ABX-MA1 was purchased from ProteoGenix (PX-TA1914, lot 110,620-A01, Schiltigheim, France). Mouse IgG isotype control (31,903, lot 077189I) and mIgG1-Alexa Fluor 488 (53–4714-42, clone P3.6.2.8.1) were purchased from Thermo Fisher Scientific (Oslo, Norway). Ultra-LEAF™ purified human IgG1 isotype control, recombinant, hIgG1, was purchased from Nordicbiosite (403,502, clone QA16A12 Kristiansand, Norway). Antibodies were labeled using the Alexa Fluor 488 (Alexa 488) Protein Labeling Kit from Thermo Scientific (A10235, Oslo, Norway), according to the manufacturer’s protocol. The secondary antibody goat anti-human Alexa 488 was purchased from Thermo Scientific (A11013, lot 2,273,669, Oslo, Norway). Antibody labeling with ^212^Pb is described below.

### Cell lines

The human mesothelioma cell lines MSTO-211H and NCI-H226 obtained from ATCC (ATCC CRL-2081 and CRL-5826, respectively) (Virginia, USA) were used in this study. The cell lines were established from the pleural effusion of patients with biphasic mesothelioma and squamous cell carcinoma, respectively. The cells were cultured in 10% complete cell medium, RPMI 1640 (Fisher Scientific, Oslo, Norway) supplemented with 10% heat-inactivated fetal bovine serum (FBS) (Fisher Scientific, Oslo, Norway) and 1% penicillin–streptomycin (Fisher Scientific, Oslo, Norway) in an incubator at 37 °C with 5% CO_2_. At 80–90% confluence, the cells were propagated and harvested by brief detachment with TrypLe Express (Fisher Scientific, Oslo, Norway) and centrifuged at 1200 rpm for 5 min.

### Flow cytometry for analysis of CD146 expression on MM cell lines

MM cell lines were harvested, washed and cell number and viability were determined using a Countess Cell Counter (Invitrogen, Carlsbad, USA), followed by dilution in a flow cytometry buffer, Dulbecco’s PBS (VWR, Oslo, Norway) with 0.5% BSA (VWR, Oslo, Norway). The cell suspension was adjusted to 1 × 10^6^ cells/tube. Between 0.001–10 µg of Alexa 488-labeled antibodies was added to the tubes and incubated at 4 °C for 30 min before washing the cells with 500 µL flow buffer. Two washing steps were performed, and the cells were centrifuged at 1200 rpm for 5 min.

For saturation experiments, Alexa 488-labeled antibodies were added to 0.2 × 10^6^ cells/tube at a concentration of 100–0.001 nM and incubated at 4 °C for 3 h before washing the cells twice with 500 µL of the flow buffer.

Additionally, all cells were stained with MitoTracker Red CMXRos (Thermo Scientific, Oslo, Norway) for 30 min to specifically label mitochondria in live cells and then washed twice with flow buffer. MitoTracker Red CMXRos is a cell-permeable reagent used for labeling the mitochondria in live cells^61^. The washed cell pellets were dissolved in 100 µL of flow buffer with 1 µM DRAQ5 (Fisher Scientific, Oslo, Norway) added 10 min before the samples were collected on a flow cytometer to exclude dead cells. Draq5 has high affinity for double-stranded DNA in live cells^62^. The samples were collected using a CytoFLEX Flow Cytometer (Beckman Coulter, Indianapolis, USA). The samples were collected in triplicate. Experiments were repeated at least twice or thrice for both cell lines on independent days. Data were analyzed using CytExpert version 2.4 (Beckman Coulter, Indianapolis, USA). Briefly, gating was performed on singlets, followed by specific gating on MitoTracker Red CMXRos and DRAQ5 double-positive populations to exclude dead cells. This type of staining was used in all the flow cytometry experiments (described below). For statistical analysis, the relative median fluorescence intensity (rMFI) was calculated compared to unstained cells (rMFI sample – rMFI unstained)/rMFI unstained, and the mean with standard deviation was calculated for samples in triplicate for each experiment. For the final graphs, the average and standard deviation were calculated from multiple experiments. For the saturation experiments, a one-site binding model (Y = Bmax × X / (K_D_ + X)) was used to analyze the data plotted as a hyperbolic curve using GraphPad Prism, version 10.1.2 (La Jolla, USA). This analysis aimed to determine the apparent equilibrium dissociation constants (K_D_^APP^) of mOI-3 and chOI-3 in the MSTO-211H and NCI-H226 cells.

To assess different cell-surface stripping buffers, 1 × 10^6^ NCI-H226 cells/tube were incubated with 0.25 µg Alexa 488 labeled ChOI-3 for 30 min at 4ºC and washed thoroughly twice with Dulbecco’s PBS. Four cell-surface stripping buffers were tested: 1. 50 mM glycine (VWR, Oslo, Norway), 150 nM NaCl (VWR, Oslo, Norway) pH 2.6 for 10 min^51^, 2. 1% citric acid (VWR, Oslo, Norway), 0.5 M NaCl, pH 3 for 45 min^63^, 3. 1% w/v pepsin diluted 1:100 (Sigma Aldrich, Oslo, Norway), in PBS pH 3–4 for 45 min^64^ and 4. 0.01 g/mL trypsin (Sigma Aldrich, Oslo, Norway), in PBS pH 9 for 60 min^64^. The cells were kept in the buffer for the indicated time points at 37 °C in an orbital shaker. Cells were washed twice with flow buffer at the end of the incubation period before being evaluated for the Alexa 488 signal on a flow cytometer and compared to chOI-3 Alexa 488, isotype control, and unstained cells. For each condition, the rMFI was calculated from triplicate samples for each experiment. The percentage of collected events was depicted as the percentage of the total number of events collected on a flow cytometer. From the collected events for samples in triplicate, the percentage of MitoTracker Red CMXRos and DRAQ5 double-positive populations was calculated.

### Affinity measurements by surface plasmon resonance

Surface Plasmon Resonance (SPR) investigations of mOI-3 and chOI-3 were performed by Abzena (Cambridge, UK) using multicycle kinetics in a Biacore T2000 system. The antibodies were immobilized on a CM5 chip coupled with either anti-mouse IgG or anti-human IgG with  ~ 9000 RU. The antibodies were diluted in running buffer (HBS-EP and 1 mg/mL BSA). The analyte was human CD146 protein (SinoBiological, Eschborn, Germany) at a 7-point twofold dilution from 180 nM to 2.8 nM. The Biacore sensor chip was immobilized with a mouse capture antibody and a human capture antibody to run both mouse and chimeric antibodies in the same experiment. In experiments with chimeric antibodies alone (chOI-3 and chOI-3-TCMC-conjugated antibodies), recombinant protein A/G variants were pre-immobilized onto the sensor chip surface to capture the antibody^65^. The equilibrium dissociation constant (K_D_) was calculated as K_d_/K_a_, where K_d_ and K_a_ are the measured dissociation and association constants, respectively.

### Evaluation of cellular internalization by flow cytometry

The surface binding and internalization of mOI-3 and chOI-3 in MSTO-211H and NCI-H226 cells were assessed by flow cytometry using Alexa 488 labeled antibodies. For the internalization assay, 0.35 × 10^6^ MSTO-211H or NCI-H226 cells/well were seeded on the day of the experiment in 24-well plates (Corning, NY, USA) in 10% complete cell media. Alexa 488 conjugated mAb (0.25 µg) was added to the cells and incubated for the indicated periods of time at 37 °C on an orbital shaker. 30–45 min before the end of incubation, cells were labeled with MitoTracker Red CMXRos. At a given time point, the culture medium was removed, and cells were washed twice with DPBS before adding a stripping buffer for these cells to samples, trypsin, 0.01 g/mL in PBS, pH 8–9, (Sigma Aldrich, Oslo, Norway) for 1 h, similar to the method of Lin et al.^64^. Following stripping, the cells were washed twice with the flow buffer prior to analysis. Flow cytometry was performed on non-stripped and stripped cell samples to assess the change in the Alexa 488 signal under the described conditions for each time point (1-, 6- and 24-h). The cells were analyzed using a flow cytometer to determine the degree of receptor internalization in comparison to the total signal. Surface binding was assessed as rMFI, as previously described. The percentage of internalized signal was calculated as internalized rMFI signal (trypsin-stripped cells) compared to the total-bound rMFI (non-stripped cells), rMFI_Internalized_/rMFI_total_ × 100%.

### Evaluation of cellular internalization by IncuCyte

MSTO-211H cells were seeded in flat-bottom clear 96-well plates (Greiner, Kremsmünster, Austria) at a seeding density of 1 × 10^3^ cells/well 24 h prior to the addition of antibodies at 37 °C with 5% CO_2_. Antibody treatments included chOI-3 and a human IgG1 antibody (BioLegend, San Diego, United States) as an isotype control, each pre-conjugated with the IncuCyte FabFluor-pH Red reagent (Sartorius, Göttingen, Germany). The fluorescence signal generated from the IncuCyte FabFluor-pH Red reagent was imaged every 30 min with nine images/well in the phase contrast and red fluorescence channels of the Incucyte Live‐Cell imaging system (S3, Sartorius, Göttingen, Germany) as a measure of internalization of the corresponding antibodies over time. For statistical analysis, the mean of the area under the curve (AUC) was calculated followed by an unpaired *t*-test using GraphPad Prism, version 10.1.2 (La Jolla, USA).

### Immunohistochemistry (IHC) staining

Immunohistochemistry was performed by Creative Bioarray (Shirley, NY, USA) to evaluate species cross-reactivity. Frozen tissue microarray slides containing healthy tissues and organs were purchased from Zyagen (San Diego, USA) for rat, (RAF-MT3), and mouse, (MAT-MT3) along with frozen human tissue microarray slides from Creative Bioarray (FRTMA057). Frozen tissue microarray slides were fixed using cold 1:1 acetone/methanol solution for 15 min before blocking with normal goat serum in PBS. This was followed by an overnight incubation at 4 °C with 1:50 dilutions of mOI-3 (for human and rat tissues) and chOI-3 (for mouse tissues). This was followed by an overnight incubation at 4 °C with 1:50 dilutions of mOI-3 (for human and rat tissues) and chOI-3 (for mouse tissues). To reduce background noise, 1% hydrogen peroxide block in PBS was applied for 15 min. The mOI-3-stained slides were then probed with VisUCyte HRP Goat Anti-Mouse polymer for 1 h at room temperature. For chOI-3 staining, the slides were incubated with a biotinylated anti-human secondary antibody for 60 min and then with streptavidin-HRP for 30 min at room temperature. Sections were developed with DAB solution for varying times: FRTMA057 for 4 min, RAF-MT3 for 3 min, and MAT-MT3 for 1 min, tailored to the individual sample requirements. Following the DAB reaction, the slides were further contrasted with a 40 s Mayer’s hematoxylin counterstain before being dehydrated, cleared, and mounted. The staining was reviewed and imaged using an Olympus automatic slide scanner, and images were processed using the appropriate software. The slides were analyzed and scored by a pathologist. The H scoring system is based on a scale from 0 (no signal) to + 3 (strong signal).

### Preparation of ^212^Pb and radioactivity measurements

Lead-212 was produced by ^220^Rn emanation from ^228^Th obtained from Oak Ridge National Laboratory (Oak Ridge, USA), or ^224^Ra solution prepared as previously described^57^. A simplified single-chamber system was used, containing a 100 mL glass flask turned upside down and a removable cap with a radionuclide source^66^. The ^228^Th in 1 M HCl was applied to a piece of quartz wool acting as holding material inside the cap. During decay, ^220^Rn emanated from the holding material and was adsorbed into the interior of the flask as ^212^Pb. After 1–2 days of decay, the flask was carefully removed from the source cap and rinsed with 0.1 M HCl to collect the deposited ^212^Pb.

Lead-212 activity was determined by measuring radioactive samples on a Hidex Automatic Gamma Counter (Hidex Oy, Turku, Finland) with a 60–110 keV counting window and Capintec CRC-25R radioisotope dose calibrator (Capintec Inc. Ramsey, NJ, USA) with a calibration number of 662, a dial setting specifically established for the instrument used^67^.

### Radiolabeling and quality control of antibodies with ^212^Pb

Antibodies mOI-3, chOI-3, mIgG1, and hIgG1 in carbonate buffer were conjugated with a 5–20-fold molar excess of S-2-(4-isothiocyanatobenzyl)-1,4,7,10-tetraaza-1,4,7,10-tetra(2-carbamoylmethyl) cyclododecane (p-SCN-Bn-TCMC, TCMC; Macrocyclics Inc, Dallas, USA)) in 5 mM HCl (Merck, Darmstadt, Germany) at room temperature for 2 h. Unbound TCMC was removed by exchanging carbonate buffer with 0.9% NaCl (Merck, Darmstadt, Germany). The extracted ^212^Pb in 0.1 M HCl obtained from the emanation generator was adjusted to pH 5–6 with 5 M sodium acetate (Merck, Darmstadt, Germany) and mixed with TCMC-mAbs with specific activities of 1–50 MBq/mg. The solution was incubated for 30–35 min in a Thermomixer (Eppendorf, Oslo, Norway) at 37 °C with shaking at 350 rpm and then diluted in a formulation buffer consisting of DPBS with 7.5% (v/v) recombinant albumin (Octapharma, Lachen, Switzerland), 200 mM sodium ascorbate (Sigma-Aldrich, Oslo, Norway), and 1 mM EDTA (Sigma-Aldrich, Oslo, Norway) at pH 7.4, resulting in ^212^Pb-TCMC-mAb. The radiochemical purity of the final products was assessed using chromatography strips from Biodex (Shirley, NY, USA) and gamma counting as previously described^57^. All the ^212^Pb conjugates used in this study had a radiochemical purity of > 96%.

To quickly and easily evaluate the immunoreactive fractions (IRF) of the radiolabeled antibodies, an IRF assay using frozen stocks of the high CD146 antigen-positive osteosarcoma cell line, OHS^68^, was established. IRF was measured using one-point binding assays *in vitro* as previously described^14^. Briefly, a single cell suspension, ranging from 10–15 × 10^6^ cells, was prepared in Dulbecco’s PBS supplemented with 0.5% bovine serum albumin (BSA). Triplicate samples, each consisting of 0.2 mL of cell solution, were incubated at room temperature for approximately 60 min with gentle shaking, in the presence of 2 ng ^212^Pb-TCMC-mAb. To estimate non-specific binding, the samples were preincubated for 15–30 min with 20 µg of unlabeled mAb before the addition of the radiolabeled mAb. The total radioactivity in each sample was measured using Hidex automatic gamma counter. Following centrifugation and washing of the cells three times with Dulbecco’s PBS containing 0.5% BSA, cell-bound radioactivity was measured in the cell pellets. The fraction of bound mAb was calculated by dividing the amount of cell-bound activity by the total added radioactivity, and immunoreactivity was determined by subtracting the fraction of nonspecifically bound mAb from the fraction of bound mAb.

### Binding and internalization of ^212^Pb-TCMC-mAb into MSTO-211H

The ^212^Pb-TCMC-mAb was used to quantitatively assess internalization in MSTO-211H cells, following a method similar to Heyerdahl’s^69^. After preparing the cells as described in the flow cytometry internalization assay above, the cells underwent identical washing and stripping steps using 0.01 g/mL trypsin in PBS, pH 9, following the addition of ^212^Pb-TCMC-mAb (0.5 µg/mL) at 2- and 24-h time points. ^212^Pb-TCMC-chOI-3 and the negative control ^212^Pb-TCMC-hIgG1 were incubated with the cells for the indicated time. For the quantitative assessment of internalization, radioactivity in the wash solutions (cell surface stripped fraction) and the stripped cell pellet (internalized fraction) was measured using a Hidex automatic gamma counter. Radioactivity measurements were background- and decay-corrected. The assay was not corrected for non-specific binding; however, the cells were treated with 0.5% BSA 30 min before the addition of ^212^Pb-TCMC-mAb to reduce non-specific binding. The amounts of ^212^Pb and ^212^Bi atoms in different fractions were calculated to determine the total binding (internalized and surface-bound), internalized, and surface-bound fractions. Fold change in internalization was calculated as the number of ^212^Pb or ^212^Bi atoms internalized by chOI-3 divided by internalized atoms hIgG, i.e. atom_internalized chOI-3_/atom_internalized hIgG_. The final result was presented as %internalized, i.e., the ratio of the internalized radioactivity to the total bound radioactivity. The activity of ^212^Bi was indirectly assessed by measuring the gamma radiation from its daughter nuclide ^208^Tl within a 520–640 keV window using a Hidex gamma counter conducted 20 min after reaching transient equilibrium between ^208^Tl and ^212^Bi^57^. The amount of ^212^Bi in Bq was calculated using the equilibrium constant for ^208^Tl/^212^Bi = 0.361 such that the measured ^208^Tl Bq / 0.361 =^ 212^Bi Bq^70^. The equilibrium between ^212^Bi and ^208^Tl upon measurement is critical to ensure an accurate activity assessment, which is particularly important in scenarios where significant isotope translocation could influence the results. To estimate the number of ^212^Pb and ^212^Bi atoms, the formula *A* = λ*N* was used, where *A* is the activity in becquerels, λ is the decay constant, and *N* is the number of atoms. The average surface and internalized number of atoms per cell were calculated for two independent experiments. Additionally, the rate of internalization of ^212^Pb and ^212^Bi atoms per cell was calculated by observing the fold change from 2 to 24 h compared to the isotype control.

### Animals

All animal-related procedures adhered to Norwegian and EU guidelines for animal research, as well as the ARRIVE (https://arriveguidelines.org/) guidelines to ensure comprehensive and transparent reporting of the study. This study was approved by the Institutional Committee on Research Animal Care of the Department of Comparative Medicine, Oslo University Hospital (Oslo, Norway), and the Norwegian Food Safety Authority (FOTS permit ID: 23,766). *In vivo* studies were conducted using female nude athymic mice (Hsd: Athymic Nude-Foxn1^nu^) aged approximately six to seven weeks, sourced from the Department of Comparative Medicine at the Norwegian Radium Hospital, Oslo University Hospital (Oslo, Norway). A total of 55 animals were used.

### *In vivo* therapeutic efficacy in xenograft mouse model with intraperitoneal mesothelioma

The therapeutic effect of radiolabeled mOI-3 was evaluated and compared with that of the radiolabeled mouse isotype control by evaluating the survival rate of mice with intraperitoneal (i.p.) mesothelioma xenografts. Tumors were established in 35 female nude mice aged 4–5 weeks by inoculation with 2.5 × 10^6^ MSTO-211H cells. In a previous study, the number of inoculated cells and rate of tumor growth were determined in a pilot animal study conducted to establish this animal model (data not shown). Five days after cell inoculation, the mice were treated to mimic a clinical scenario of tumor buildup, with the aim of achieving a targeted therapeutic effect. The mice were randomized into five different groups, with seven mice per group, and each mouse received an i.p. bolus of either 1) saline, 2) mOI-3, 3) mIgG, 4) 348 kBq ^212^Pb-TCMC-mOI-3, or 5) 371 kBq ^212^Pb-TCMC-mIgG (5 × TCMC/Ab ratio for both mOI-3 and mIgG) with a constant antibody amount of 10 µg per mouse for groups 2–5. Mice were observed during the course of the study by monitoring their body weight, activity level, posture, and i.p. tumor development every 2–3 days. All animals were first anesthetized with sevoflurane, then euthanized by cervical dislocation exclusively at disease-related endpoints, which included 10% weight loss over 1 week, low activity, hunched posture, and clinical signs of tumor burden. Changes in weight were monitored and recorded (Fig. S3). Tumor size measurement was not performed in this study due to the diffuse and nodular distribution of the tumor, which manifests as dispersed clusters of white nodules throughout the peritoneal cavity, adhering to the cavity wall and between organs. This distribution makes accurate collection and measurement challenging, precluding reliable quantification of tumor size. Animals were censored if they lived beyond the time point corresponding to three times the median survival time of the longest surviving group.

Statistically significant differences between the resulting survival curves were evaluated using GraphPad Prism software (version 10.1.2, La Jolla, USA). The Gehan–Breslow Wilcoxon method was employed, and the obtained *p*-values were adjusted using the Holm–Sidak method for multiple comparisons, with a significance threshold of p_adj_ < 0.05.

## Supplementary Information

Supplementary Information.

## Data Availability

The data that support the findings of this study are available from Oncoinvent ASA, but restrictions apply to the availability of these data, which were used under license for the current study, and so are not publicly available. Data are however available from the corresponding author at lindland@oncoinvent.com upon reasonable request and with permission of Oncoinvent ASA.
