# Supplementary material for: Antigen targeting and anti-tumor activity of a novel anti-CD146 212Pb internalizing alpha-radioimmunoconjugate against malignant peritoneal mesothelioma
Source: Sci Rep. 2024 Oct 29;14:25941. doi: 10.1038/s41598-024-76778-z (PMC11522520; doi:10.1038/s41598-024-76778-z)
Supplement: Supplementary file 1 — Supplementary Information. [file 41598_2024_76778_MOESM1_ESM.pdf]

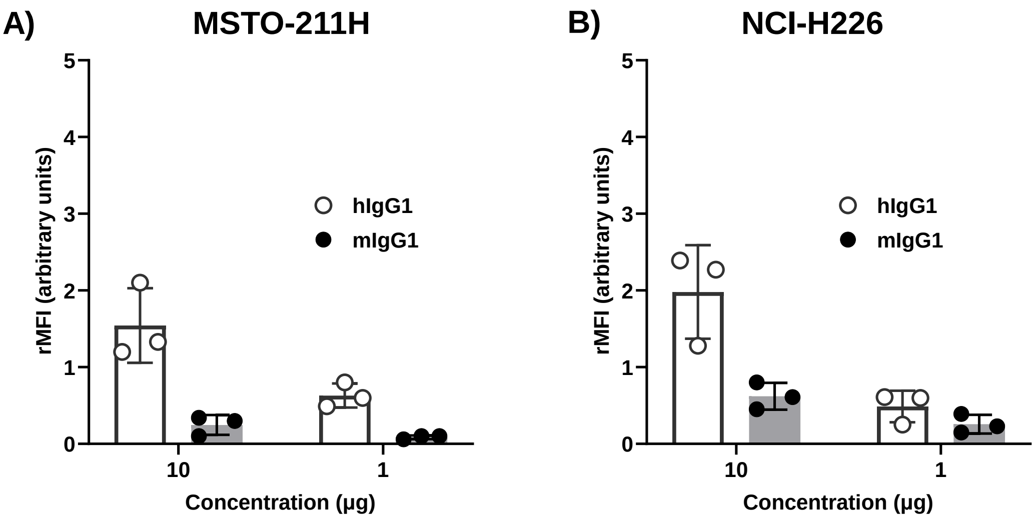

Fig S1. Isotype control, hIgG1 and mIgG1, show low binding to MSTO-211H and NCI-H226 cells. A) MSTO-211H and B) NCI-H226 cells were stained with 1 or 10 µg of hIgG1 Alexa 4888 or mIgG1 Alexa 488 for 3 hours at 4 °C. Only MitoTracker Red CMXRos and DRAQ5 double positive cells were included in analysis. Relative median fluorescence intensity (rMFI) for triplicate samples was calculated based on MFI of unstained cells for each experiment. Each point represents mean of triplicate rMFI with SD. Data are representative as mean ±SD from three independent experiments.

A)

Range 1: 1 to 648 GenPept Graphics ▼ Next Match ▲ Previous Match

Score	Expect	Method	Identities	Positives	Gaps
1011 bits(2613)	0.0	Compositional matrix adjust.	494/648(76%)	567/648(87%)	2/648(0%)

```
Query  1    MGLPRLVCAFLLAACCCCPRVAGVPGEAEQPAP--ELVEVEVGSTALLKCGLSQSQGNLS  58
            MGLP+LVC FL AACCCC R AGVPGE +QP P  +LVE EVGSTALLKCG S++ GN S
Sbjct  1    MGLPKLVCVFLFAACCCCRRAAGVPGEEKQPVPTPDLVEAEVGSTALLKCGPSRASGNFS  60

Query  59   HVDWFSVHKEKRTLIFRVRQGQGQSEPGEYEQRLSLQDRGATLALTQVTPQDERIFLCQG  118
             VDWF +HKE++ LIFRV QG+GQ EPGEYE RLSLQD  ATLAL+ VTP DER+FLC+
Sbjct  61   QVDWFLIHKERQILIFRVHQGKGQREPGEYEHRLSLQDSVATLALSHVTPHDERMFLCKS  120

Query  119  KRPRSQEYRIQLRVYKAPEEPNIQVNPLGIPVNSKEPEEVATCVGRNGYPIPQVIWYKNG  178
            KRPR Q++ ++L+V+KAPEEP IQ N +GI V+ +E  EVATCVGRNGYPIPQV+WYKN
Sbjct  121  KRPRLQDHYVELQVFKAPEEPTIQANVVGIHVDRQELREVATCVGRNGYPIPQVLWYKNS  180

Query  179  RPLKEEKNRVHIQSSQTVESSGLYTLQSILKAQLVKEDKDAQFYCELNYRLPSGNHMKES  238
             PL+EE+NRVHIQSSQ VESSGLYTL+S+L A+LVKEDKDAQFYCEL+YRLPSGNHMKES
Sbjct  181  LPLQEEENRVHIQSSQIVESSGLYTLKSVLSARLVKEDKDAQFYCELSYRLPSGNHMKES  240

Query  239  REVTVPVFYPTEKVWLEVEPVGMLKEGDRVEIRCLADGNPPPHFSISKQNPSTREAEEET  298
            +EVTVPVFYP EKVW+EVEPVG+LKEGD V IRCL DGNP PHF+I+K++PST E EEE+
Sbjct  241  KEVTVPVFYPAEKVWVEVEPVGLLKEGDHVTIRCLTDGNPQPHFTINKKDPSTGEMEEES  300

Query  299  TNDNGVLVLEPARKEHSGRYECQGLDLDTMISLLSEPQELLVNYVSDVRVSPAAPERQEG  358
            T++NG+L LEPA K HSG Y+CQ LDL+T I+L S+P ELLVNYVSDV+V+P APE QEG
Sbjct  301  TDENGLLSLEPAEKHHSGLYQCQSLDLETTITLSSDPLELLVNYVSDVQVNPTAPEVQEG  360

Query  359  SSLTLTCEAESSQDLEFQWLREETGQVLERGPVLQLHDLKREAGGGYRCVASVPSIPGLN  418
             SLTLTCEAES+QDLEF+WLR++TGQ+L +GPVLQL++++REAGG Y C+ASVP +PGLN
Sbjct  361  ESLTLTCEAESNQDLEFEWLRDKTGQLLGKGPVLQLNNVRREAGGRYLCMASVPRVPGLN  420

Query  419  RTQLVNVAIFGPPWMAFKERKVWVKENMVLNLSCEASGHPRPTISWNVNGTASEQDQDPQ  478
            RTQLV+V IFG PWMA KERKVWV+EN VLNLSCEASGHP+PTISWNVNG+A+E + DPQ
Sbjct  421  RTQLVSVGIFGSPWMALKERKVWVQENAVLNLSCEASGHPQPTISWNVNGSATEWNPDPQ  480

Query  479  RVLSTLNVLVTPELLETGVECTASNDLGKNTSILFLELVNLTTLTPDSNTTTGLSTSTAS  538
             V+STLNVLVTPELLETG ECTASN LG NT+ + L+LV LTTL PDS+ TTGLST T S
Sbjct  481  TVVSTLNVLVTPELLETGAECTASNSLGSNTTTIVLKLVTLTTLIPDSSQTTGLSTLTVS  540

Query  539  PHTRANSTSTERKLPEPESRGVVIVAVIVCILVLAVLGAVLYFLYKKGKLPCRRSGKQEI  598
            PHTRANSTSTE+KLP+PES+GVVIVAVIVC LVLAVLGA LYF YKKGKLPC RSGKQEI
Sbjct  541  PHTRANSTSTEKKLPQPESKGVVIVAVIVCTLVLAVLGAALYFFYKKGKLPCGRSGKQEI  600

Query  599  TLPPSRKSELVVEVKSDKLPEEMGLLQGSSGDKRAPGDQGEKYIDLRH  646
            TLPP+RKSE VVEVKSDKLPEEM LLQGS+GDKRAPGDQGEKYIDLRH
Sbjct  601  TLPPTRKSEFVVEVKSDKLPEEMALLQGSNGDKRAPGDQGEKYIDLRH  648
```

B)

Range 1: 1 to 600 GenPept Graphics ▼ Next Match ▲ Previous Match

Score	Expect	Method	Identities	Positives	Gaps
896 bits(2315)	0.0	Compositional matrix adjust.	443/600(74%)	520/600(86%)	2/600(0%)

```
Query  1    MGLPRLVCAFLLAACCCCPRVAGVPGEAEQPAP--ELVEVEVGSTALLKCGLSQSQGNLS  58
            MGLPRLVCAFL AACCCC    GVPGE +QP P  + VEVEVG+TALLKCG +   GN S
Sbjct  1    MGLPRLVCAFLFAACCCCRSATGVPGEEKQPTPTPDPVEVEVGNTALLKCGPAHPSGNFS  60

Query  59   HVDWFSVHKEKRTLIFRVRQGQGQSEPGEYEQRLSLQDRGATLALTQVTPQDERIFLCQG  118
             V+WF +HKE++  IFRV QG+GQSEPGEYE RLSL   GATLAL+QVTP D+R+FLC+
Sbjct  61   QVEWFLIHKERQIPIFRVHQGKGQSEPGEYEHRLSLHGPGATLALSQVTPHDDRMFLCKS  120

Query  119  KRPRSQEYRIQLRVYKAPEEPNIQVNPLGIPVNSKEPEEVATCVGRNGYPIPQVIWYKNG  178
            K+PR Q++ +QL+VYKAPEEP IQ N LGI V+ +E +EVATCVGRNGYPIPQVIWYKNG
Sbjct  121  KQPRPQDHYVQLQVYKAPEEPTIQANVLGIHVDIQELKEVATCVGRNGYPIPQVIWYKNG  180

Query  179  RPLKEEKNRVHIQSSQTVESSGLYTLQSILKAQLVKEDKDAQFYCELNYRLPSGNHMKES  238
            RPL+EE+NRVHIQSSQTVESSGLYTL+S+L A+LVKEDKDAQFYCEL+YRLPSGN MKES
Sbjct  181  RPLQEEENRVHIQSSQTVESSGLYTLKSVLSARLVKEDKDAQFYCELSYRLPSGNRMKES  240

Query  239  REVTVPVFYPTEKVWLEVEPVGMLKEGDRVEIRCLADGNPPPHFSISKQNPSTREAEEET  298
            +EVTVPV YP EKVW+EVEPVG+LKEGD V+IRCL DGNP PHF+I+K+NPST E EEE+
Sbjct  241  KEVTVPVLYPAEKVWVEVEPVGLLKEGDHVKIRCLTDGNPQPHFTINKKNPSTEEMEEES  300

Query  299  TNDNGVLVLEPARKEHSGRYECQGLDLDTMISLLSEPQELLVNYVSDVRVSPAAPERQEG  358
            T++NG+L LEPA+K HSG Y+CQ LDL+T + L S+P ELLVNYVSDV+V P APE QEG
Sbjct  301  TDENGLLSLEPAQKHHSGVYQCQSLDLETTVMLSSDPLELLVNYVSDVQVDPTAPEVQEG  360

Query  359  SSLTLTCEAESSQDLEFQWLREETGQVLERGPVLQLHDLKREAGGGYRCVASVPSIPGLN  418
             SLTLTC+AES+QDLEF+WLR++TGQ+L +GP+LQL+++KREAGG Y CVASVPS+PGLN
Sbjct  361  DSLTLTCKAESNQDLEFEWLRDKTGQLLGKGPILQLNNVKREAGGRYLCVASVPSVPGLN  420

Query  419  RTQLVNVAIFGPPWMAFKERKVWVKENMVLNLSCEASGHPRPTISWNVNGTASEQDQDPQ  478
            RT+ V+V IFG PWMA KERKVW +EN +LNLSCEASGHP+PTISWN+NG+A+E + DPQ
Sbjct  421  RTRRVSVGIFGSPWMAAKERKVWAQENAMLNLSCEASGHPQPTISWNINGSATEWNPDPQ  480

Query  479  RVLSTLNVLVTPELLETGVECTASNDLGKNTSILFLELVNLTTLTPDSNTTTGLSTSTAS  538
             V+STLNVLVTPELLETG ECTASN LG NT+++ L+LV LTTLTPDS+ TTGLST T S
Sbjct  481  TVVSTLNVLVTPELLETGAECTASNSLGSNTTVIILKLVTLTTLTPDSSQTTGLSTPTVS  540

Query  539  PHTRANSTSTERKLPEPESRGVVIVAVIVCILVLAVLGAVLYFLYKKGKLPCRRSGKQEI  598
            PH+RANSTSTE+KLP+ ES+GVVIVAVIVC LVLAVLGA LY+ YKKGKLPC RSGKQE+
Sbjct  541  PHSRANSTSTEKKLPQQESKGVVIVAVIVCTLVLAVLGATLYYFYKKGKLPCGRSGKQEM  600
```

Fig S2.Protein sequence comparison between human and mouse and human and rat CD146 sequence. A) and B) NCBI Protein BLAST shows 76% and 74% protein sequence similarity between human CD146 and mouse and rat, respectively.

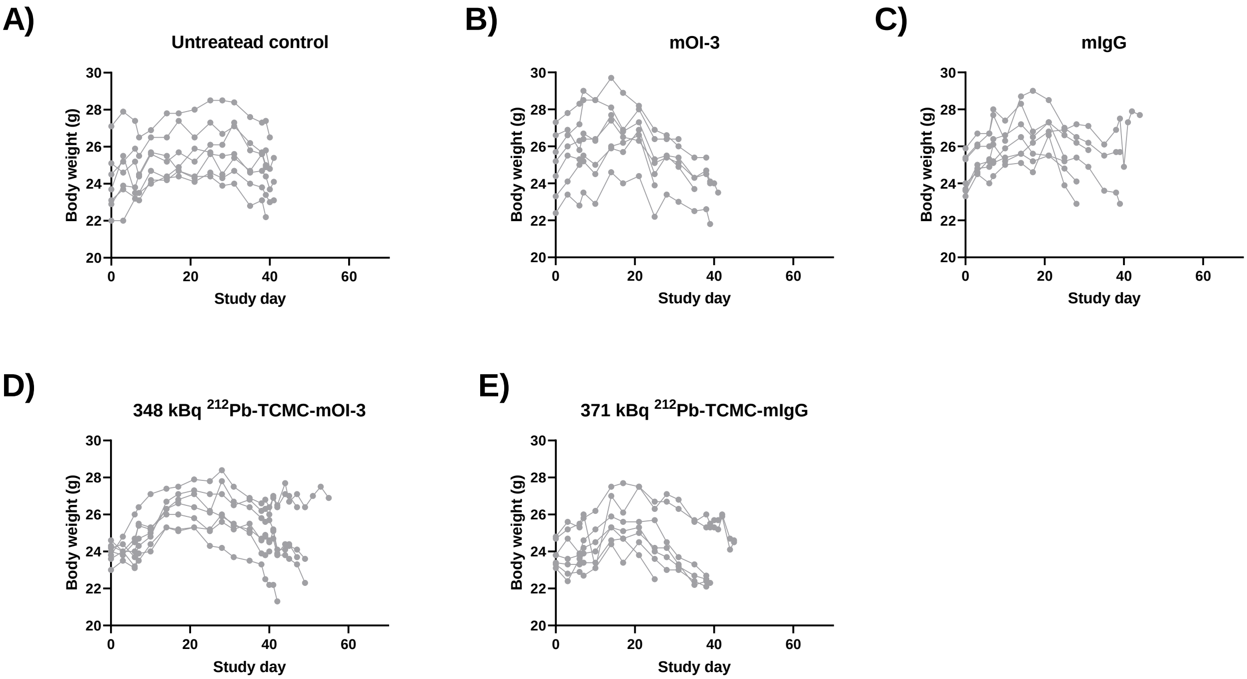

Fig S3. Body weight monitoring of animals in the 212Pb-mOI-3 study efficacy study. Body weight of n=7 nude mice per group is presented as individual weight for every mouse. Nude mice were inoculated intraperitoneally with MSTO-211H cells on day zero and treated intraperitoneally on day 6 with : A) Saline, B) mOI-3, and C) mIgG, D) 348 kBq ^{212}Pb-TCMC-mOI-3, and E) 371 kBq ^{212}Pb-TCMC-mIgG. In groups B-E, the antibody amount was 10 µg.
